# Influence of Auditory Cues on the Neuronal Response to Naturalistic Visual Stimuli in a Virtual Reality Setting

**DOI:** 10.3389/fnhum.2022.809293

**Published:** 2022-06-02

**Authors:** George Al Boustani, Lennart Jakob Konstantin Weiß, Hongwei Li, Svea Marie Meyer, Lukas Hiendlmeier, Philipp Rinklin, Bjoern Menze, Werner Hemmert, Bernhard Wolfrum

**Affiliations:** ^1^Neuroelectronics – Munich Institute of Biomedical Engineering, Department of Electrical and Computer Engineering, Technical University of Munich, Munich, Germany; ^2^Department of Quantitative Biomedicine, University of Zurich, Zurich, Switzerland; ^3^Department of Informatics, Technical University of Munich, Munich, Germany; ^4^Bio-Inspired Information Processing – Munich Institute of Biomedical Engineering, Department of Electrical and Computer Engineering, Technical University of Munich, Munich, Germany

**Keywords:** brain computer interface, event-related potential (ERP), combinational audio-visual stimulus, visual evoked potential (VEP), virtual reality, support vector machine (SVM)

## Abstract

Virtual reality environments offer great opportunities to study the performance of brain-computer interfaces (BCIs) in real-world contexts. As real-world stimuli are typically multimodal, their neuronal integration elicits complex response patterns. To investigate the effect of additional auditory cues on the processing of visual information, we used virtual reality to mimic safety-related events in an industrial environment while we concomitantly recorded electroencephalography (EEG) signals. We simulated a box traveling on a conveyor belt system where two types of stimuli – an exploding and a burning box – interrupt regular operation. The recordings from 16 subjects were divided into two subsets, a visual-only and an audio-visual experiment. In the visual-only experiment, the response patterns for both stimuli elicited a similar pattern – a visual evoked potential (VEP) followed by an event-related potential (ERP) over the occipital-parietal lobe. Moreover, we found the perceived severity of the event to be reflected in the signal amplitude. Interestingly, the additional auditory cues had a twofold effect on the previous findings: The P1 component was significantly suppressed in the case of the exploding box stimulus, whereas the N2c showed an enhancement for the burning box stimulus. This result highlights the impact of multisensory integration on the performance of realistic BCI applications. Indeed, we observed alterations in the offline classification accuracy for a detection task based on a mixed feature extraction (variance, power spectral density, and discrete wavelet transform) and a support vector machine classifier. In the case of the explosion, the accuracy slightly decreased by –1.64% p. in an audio-visual experiment compared to the visual-only. Contrarily, the classification accuracy for the burning box increased by 5.58% p. when additional auditory cues were present. Hence, we conclude, that especially in challenging detection tasks, it is favorable to consider the potential of multisensory integration when BCIs are supposed to operate under (multimodal) real-world conditions.

## Introduction

Neuroscientists aim to understand the human brain by deciphering neuronal signals due to different tasks and stimuli (Adrian and Yamagiwa, 1935; Gross, 1999; Finger, 2001; Strotzer, 2009). Although there are other techniques, most research up to date is based on non-invasive electroencephalography (EEG) recordings, where the electrical activity across the scalp is monitored using distributed electrode arrays (Adrian and Yamagiwa, 1935; Homan et al., 1987; Cincotti et al., 2008; Nicolas-Alonso and Gomez-Gil, 2012). In the past, extensive research focused on unraveling basic neuronal patterns in response to different isolated conditions (Adrian and Yamagiwa, 1935; Penfield and Evans, 1935; Davis et al., 1939; Davis, 1939; Hill, 1958). Thus, an extensive collection of experimental paradigms that evoke specific responses – e.g., event-related potentials (ERPs), steady-state visually evoked potentials (SSVEPs), and motor imaginary related activity, among others – has been established (Ritter et al., 1979; Lines et al., 1984; Alho et al., 1994; Creel, 1995; Comerchero and Polich, 1999; Stige et al., 2007; Sur and Sinha, 2009). Nowadays, applied neuroscientists and engineers use these stimuli–response relations to design brain-computer interfaces (BCIs) that can automatically read out and analyze signals for a specific task. For instance, the P300-speller, a brain-controlled wheelchair, and a brain-controlled prosthetic arm are common BCI applications in the medical context (Rebsamen et al., 2010; Belitski et al., 2011; Nicolas-Alonso and Gomez-Gil, 2012; Abdulkader et al., 2015; Bright et al., 2016). Furthermore, recent technological improvements enable EEG recordings not only under “clean” laboratory conditions but also in natural environments via portable EEG devices. Hence, there is considerable interest in translating BCI applications into more complex real-world settings (Zander and Kothe, 2011). However, in such scenarios, the performance of BCIs and their discriminatory power are drastically affected by interfering signals and physiological artifacts (Fatourechi et al., 2007; Zander et al., 2010; Minguillon et al., 2017). Here, a combined read-out of multiple cues and/or measurement modalities – a so-called hybrid BCI (hBCI) – addresses this issue by providing an enlarged dataset for classification (Allison et al., 2010; Pfurtscheller et al., 2010; Leeb et al., 2011; Amiri et al., 2013; Yin et al., 2015; Hong and Khan, 2017). For instance, ERPs were combined with motor or mental tasks to design multiple-cue hBCIs (Hong and Khan, 2017). Additionally, parallel recordings from EEG and electrooculography (EOG) or functional near-infrared spectroscopy (fNIRS) were reported to improve performance (Amiri et al., 2013; Hong and Khan, 2017). Consequently, hBCIs offer great potential in various fields, e.g., in diagnostics, rehabilitation, machine control, entertainment, and safety (Allison et al., 2010; Blankertz et al., 2010; Brumberg et al., 2010; Nicolas-Alonso and Gomez-Gil, 2012; Hong and Khan, 2017). Another promising area of application is in the context of industry 4.0, where the aim is to operate factories most efficiently by fusing data streams and monitoring all relevant processes digitally (Douibi et al., 2021).

However, the affiliated classification tasks will be very challenging in most real-world cases depending on the paradigm and the interfering background signals. Although novel machine learning approaches help to find common patterns, they rely on massive amounts of input data. Here, virtual reality technology (VR) can help to gather consistent training data by simulating natural environments (Holper et al., 2010; Kober and Neuper, 2012; Lotte et al., 2012; Tauscher et al., 2019; Vourvopoulos et al., 2019; Marucci et al., 2021). It has been shown that VR enhances the feeling of presence and provides a real-world experience that keeps the subject more engaged (Kober and Neuper, 2012; Marucci et al., 2021). So far, most EEG-VR studies focused on 3D visual cues, disregarding the effect of simultaneous visual and acoustic stimuli in realistic situations. Previous studies on multimodal audio-visual cues, (Marucci et al., 2021) revealed that the simultaneous neuronal processing of vision and sound is strongly dependent on the exact experiment, determined by the nature, strength, and synchronicity of the stimulus.

This work aims to reveal the effect of additional auditory cues on visually-evoked ERPs within a complex naturalistic scene. To this end, we created an industrial VR environment and designed two visual stimuli that are different in the degree of event severity and stimulus strength. In our experiment, the subject’s vision is a conveyor belt-based industrial warehouse, where packages are carried along a unilateral path during regular operation. However, as we target safety applications, in some instances, the regular operation is interrupted by either an exploding or an igniting/burning box.

Since both naturalistic stimuli are visually complex, we first investigate the neuronal response to such visual stimuli and study the effect of perceived severity. Then, we compare our previous findings (visual-only) to a set of experiments, where additional auditory cues match the subject’s vision (audio-visual). Lastly, we apply three basic feature extraction methods – variance-, power-spectral-density- and discrete-wavelet-transform-based – to evaluate the effect of additional auditory cues on the classification performance by using a support vector machine (SVM) classifier. Throughout the study, we focused on hardware (24-channel portable EEG) and processing methods suitable for real-world applications.

## Materials and Methods

### Participants

Eighteen subjects (7 females, 11 males) with a mean age of 26 ± 3.4 years participated in this study. Nine subjects were recorded in a visual-only experiment, and nine participated in an audio-visual experiment. To avoid interferences and adaptation, each participant took part only in one of the two experiments. All subjects had normal or corrected to normal vision, normal hearing, no history of neurological diseases, and no previous experience with BCIs or/and EEG recordings. Subjects that exhibited a skin-to-electrode impedance above 10 kOhm across the parietal-occiptal lobe electrodes were not considered for further analysis. The study was approved by the Ethics Commission of the Technical University of Munich.

### Experimental Setup

**FIGURE 1:**
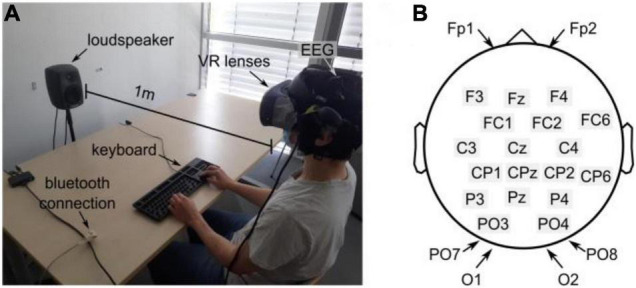
Experimental setup **(A)** Experimental environment. The subject wears a VR lens and is sitting in front of the keyboard and the loudspeaker. **(B)** EEG electrode distribution over the scalp following the 10–20 System.

The experiments were conducted in a quiet room with a mean sound pressure level (SPL) of 32.1 ± 2.1 dBA (measured with a precision sound analyzer Nor140, Norsonic-Tippkemper GmbH). All subjects were seated comfortably in an idle state in front of a keyboard, see Figure 1A. The visual scene and stimuli were designed with Blender v2.81 (The Blender Foundation) and Unity 2018 (Unity Software Inc.) and displayed via an HTC Cosmos virtual reality headset (90 FPS). In the case of an audio-visual experiment, the subjects were facing an active loudspeaker (8020C, GENELEC) placed at a distance of 1 m in front of the subject, as shown in Figure 1A.

All experiments were recorded using a portable 24-channel EEG system (SMARTING, mbraintrain, Serbia) with a sampling frequency of 250 Hz. The EEG was equipped with passive Ag/AgCl electrodes from EASYCAP (Herrsching, Germany), and a chloride-based electrogel was used (Abralyt HiCl, EASYCAP) to achieve impedance below 10 kΩ. The system featured a reference electrode (common mode sense, CMS) at FCz and a driven right leg electrode (DRL) at Fpz. All electrode locations follow the 10–20 system (see Figure 1B) and mainly covered occipital and parietal areas. The electrodes at Fp1 and Fp2 were considered to account for artifacts from eye movements.

Markers that indicate the onset of an (audio-) visual event were streamed from Unity using the lab-streaming layer for Unity asset (LSL4UNITY). Furthermore, all streams were recorded and synchronized using the SMARTING built-in streamer v3.3 for the lab-streaming layer. The data was further processed and analyzed via Matlab (Matlab and Statistics Toolbox Release 2020b, The MathWorks, Inc) combined with the toolboxes EEGlab (Delorme and Makeig, 2004) and fieldtrip (Oostenveld et al., 2011).

### Experimental Procedure and Stimulus Design

The study was divided into a visual-only and an audio-visual experiment containing additional auditory cues that matched the visual scene. In both experiments, the stimuli were simulated at the same positions in space and time during the trial. Moreover, the sequence of trials was the same for all subjects.

**FIGURE 2:**
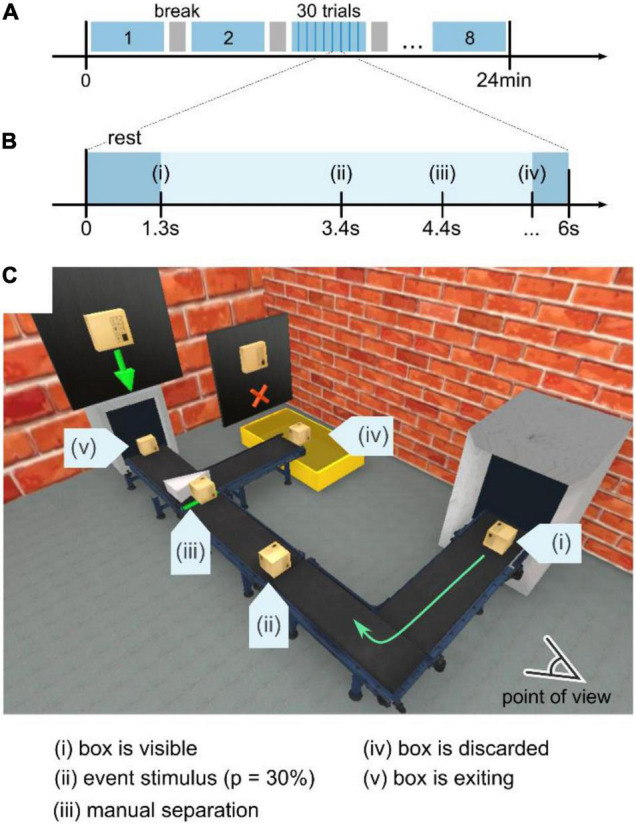
Experimental design and visual scene. **(A)** The experiment was divided into eight blocks of 4 min-recordings. In between, a subject-controlled break was implemented. The total duration of an experiment ranged from 27 to 37 min. **(B)** Each block consisted of 30 trials, either stimulus or control, with a duration of 6 s per trial. Within that period, the box traveled along the conveyor belt, as shown in panel **(C)**. A short break in between trials (between disappearance at iv and entering of a new box at i) of 1.3–2 s was implemented as visual rest time. **(C)** Visual scenery of the experiment. First, the box appears at the right part in the subject’s view (i) to subsequently move along the conveyor-belt pathway. At position (ii), the box is subject to either an explosion or ignition event (see Supplementary Material), each occurring with a probability of 15%. As the regular or ignited box travels, it reaches a junction with a manual separator, where the participant is supposed to discard the burning box and let all regular boxes pass. Exit points (iv) burning box and (v) regular box represent the spatio-temporal locations where the box disappears and the trial ends.

Each experiment (see Figure 2A) consisted of 8 blocks with a break of variable duration in between. Each block contained 30 trials with a fixed duration of 6 s per trial, as shown in Figure 2B. In general, three different conditions for the box’s pathway were implemented – either the box exploded (a), the box ignited and kept on burning (b), or the box traveled unperturbed along the pathway (c). Regardless of the trial condition, the box initially appeared in the center of the conveyor belt in the right part of the subject’s field of view (see (i), Figure 2C). Then, the box kept traveling along the conveyor belt for 2 s until it reached point (ii) in Figure 2C, where the safety-relevant events occurred with a probability of 33% (equal probability for either a burning or an exploding box) following the oddball paradigm. This probability ultimately leads to 40 stimulus trials for an exploding and 40 stimulus trials for a burning box.

The participants were told to stay seated with a visual point of view, as shown in Figure 2C. When a box appeared at point (i), the participant was instructed to track the box along the conveyor belt visually. Moreover, a short break in between trials (between disappearance at iv and entering of a new box at i) of 1.3–2 s was implemented as visual rest time.

The deviating stimuli were designed to mimic real-world scenarios, consisting of different visual characteristics (e.g., a light flash, change in size and shape). For instance, the explosion (see the video in the Supplementary Material) combined a sudden rapid change in light intensity, a swiftly propagating spherical light wave, and a disappearing flying box that occupies the entire field of view. Contrarily, in case of ignition (see the video in the Supplementary Material), the box emitted flames of fire from the center of the box. Compared to the explosion, the ignition only partially affected the scenery and started with a slower change in light intensity. While the box was traveling, the fire intensity increased until a steady state was reached.

For the burning and the control condition, the boxes were traveling past position (ii) in Figure 2C to reach the manual separator at location (iii) after 1 s. There, the subject had to manually discard the burning box toward the waste container at location (iv) by pressing the right arrow key on the keyboard. A regular box was directed to the exit (v) by pressing the up arrow key. Depending on the discarding speed, the trial duration was ∼6 s. Then, the subsequent trial started 1.3–1.5 s after the box had exited the scene at locations (iv) or (v).

In an audio-visual experiment, sounds matching the visual impressions were selected from an open-source library (freesound.org, see Supplementary Material). The sound source was attached to the traveling box in the virtual scene. However, reverberations usually stemming from walls were disabled in order to keep the acoustic scene simple. Before each experiment, the loudspeaker was adjusted to match a maximum sound level of 67 ± 0.5 dBA for the explosion and 55 ± 0.3 dBA for the burning box sound, respectively. Both sounds featured fast increasing and slowly decaying characteristics (see Supplementary Material). In the case of the burning box, the auditory cue was displayed at a constant level of 50 dBA SPL as long as the box traveled. Additionally, background noise was added to mimic a conveyor belt sound (42 ± 0.1 dBA).

### Signal Processing

**FIGURE 3:**
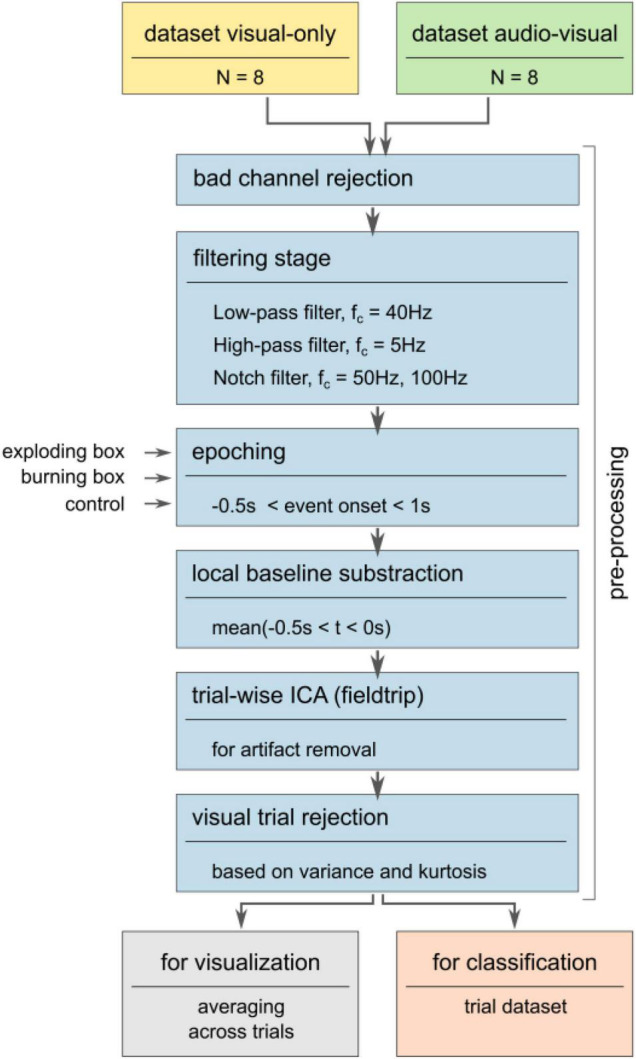
Pre-processing pipeline. The pre-processing maximizes the signal-to-noise ratio by removing bad channels and filtering the signal to a narrow frequency band. Subsequently, the signal is segmented into epochs according to markers sent from Unity. Next, the trials are visually inspected after a local baseline correction and ocular and muscle artifacts are removed via trial-based ICA. Here, high variance and/or kurtosis trials were rejected from further consideration. Then, subject-specific and global averages were computed based on a trial subset.

Eight out of the nine subjects per condition were considered while one of each group was excluded for hardware issues. The following signal processing pipeline is depicted in Figure 3. First, bad channels due to non-working electrodes were excluded. Thus, all non-working electrode were removed consistently for all participants. Then, notch filters with 50 and 100 Hz cutoff frequencies were applied to remove line noise and its second harmonic. Similar to other work, (Rozenkrants and Polich, 2008; Wang C. et al., 2012; Putze et al., 2014; Tidoni et al., 2014; Chang, 2018; Guo et al., 2019) the signal was subsequently bandpass-filtered using a low-pass FIR filter with a cutoff frequency of 40 Hz and a high-pass FIR filter with a cutoff frequency of 0.5 Hz. Consequently, all frequencies outside the narrow frequency band, such as slow drifts and high-frequency artifacts, were attenuated (Nicolas-Alonso and Gomez-Gil, 2012; de Cheveigné and Nelken, 2019). A re-referencing step was omitted due to the low number of channels and their heterogeneous distribution across the scalp (see Supplementary Figure 1). After the filter stage, the recordings were segmented into epochs according to the respective markers sent from Unity at the onset of the stimulus (position (ii) in Figures 2B,C). This segmentation resulted in a structural dataset containing all epochs ranging from t_(ii)_ –0.5s ≤ t ≤ t_(ii)_ + 1s for all three conditions, explosion (a), burning box (b), and control (c). A local baseline subtraction based on the mean signal before the onset accounted for offset differences. Then, an independent component analysis (ICA) was applied using the logistic infomax approach provided by the fieldtrip toolbox to decompose the signal (Donchin, 1966; Oostenveld et al., 2011; Chang, 2018). Subsequently, the independent components that stem from artifacts such as eye blinking and eye movement, electrode-pops, and muscle movements were visually rejected (Xue et al., 2006; Zhang et al., 2017). Here, the rejected independent component frequency spectrum and the mixing topographical matrix was inspected to decide which component was identified as an artifact. Lastly, a visual trial rejection removed trials that significantly deviated from the ensemble in terms of variance and/or kurtosis (Oostenveld et al., 2011). In general, the signal processing pipeline was established to maximize the signal-to-noise ratio and, at the same time, to avoid large signal distortions by amplification or attenuation.

In order to be consistent, subject-specific averages were computed based on 38 out of 40 stimulus trials per subject. Similarly, 38 control trials per subject were randomly selected out of 200 trials. Finally, the global responses shown in the results section were calculated as mean and standard deviation based on the subject-specific characteristics. Hence, the global average indicates the mean neuronal response of the population, whereas the standard deviation visualizes the variability between subjects. Finally, the average control condition was computed based on a random selection of 304 out of 1500 possible trials.

Finally, a statistical analysis on the difference between visual-only vs. audio-visual experiments was performed using a Welch’s *t*-test with a 5% significance level. The evaluation is based on the maximum (P1, P3b) and minimum (N2c) for each subject’s average (channel O2) and there latencies. The *t*-test assumes that both ensembles are sampled from a normally distributed dataset with unequal variance.

### Feature Extraction and Offline Classification

**FIGURE 4:**
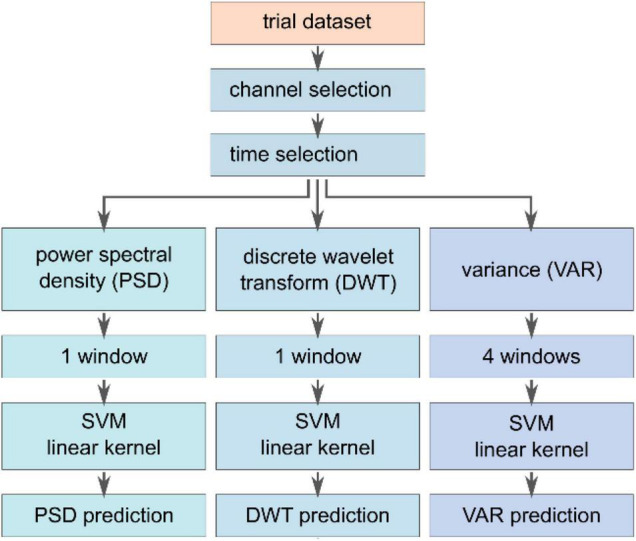
Feature extraction and classification pipeline. A subset of channels (PO3, PO4, PO8, O2, Pz, P3, P4, CPz, CP1, CP2, and Cz) was selected to compute features based on variance (VAR), power spectral density (PSD), and the discrete wavelet transform (DWT). Each feature vector was fed to an individual SVM classifier with a linear kernel. The respective vectors were normalized between –1 and 1 and concatenated to be subsequently fed into another SVM model with a linear kernel to investigate different combinations of feature vectors.

In order to assess the influence of additional auditory cues on the classification performance, different feature extraction methods, see Figure 4, – based on the variance (VAR), the power at a specific frequency band (PSD), and specific time-frequency characteristics acquired by a discrete wavelet transform (DWT) – are compared using a SVM classifier. The task of the SVM classifier was to detect the safety-relevant event – explosion (a) or ignition (b) – compared to the control condition (c), where the box was regularly traveling the pathway.

The feature extraction methods were evaluated based on the same dataset that was used for averaging. The feature vectors were computed based on channels covering the parietal and occipital lobe, namely PO3, PO4, PO8, O2, Pz, P3, P4, CPz, CP1, CP2, and Cz. Unfortunately, the channels O1 and PO7 had to be excluded due to inconsistency across subjects. The three methods were applied to the previously selected epochs for averaging with yet a smaller timeframe ranging between t_(ii)_ ≤ t ≤ t_(ii)_ + 660 ms. Each feature extraction method resulted in a dataset of feature vectors, as described in the following. The VAR method computes the variance in four different windows that have been chosen to capture the specific characteristics of the response signal, leading to a 44-element (4 values per channel, 11 channels) feature vector per trial. The first window evaluates the entire epoch from 0 ms ≤ t ≤ 660 ms, whereas the other windows split the entire interval into three successive segments of 220 ms without any overlap. Thereby, the VAR method is supposed to extract information of the entire signal and the variance of early and late potential fluctuations. The PSD feature vector of the trial was computed using the Welch-method from Matlab. Since we expect stimulus-related frequency information between 1 and 30 Hz,^38^ all other frequencies outside this window were removed, leading to a vector of length 275 (25 frequency components per channel). The third feature extraction approach, DWT relied on a Matlab discrete wavelet transform decomposition method (Bostanov, 2004; Amin et al., 2015; Cheong et al., 2015; Yahya et al., 2019). In particular, a 3-level decomposition (mother wavelet db8, window size 660 ms) was used to separate the signal in an approximate coefficient vector that extracts low-frequency information and a detail coefficient vector including the high-frequency components. The DWT vector had a length of 341 (31 approximate features per trial). The considered features were normalized and concatenated into a single vector to investigate different feature vector combinations amongst the three approaches. Here, e.g., in the case of the combined VAR-PSD-DWT feature, the vector had a length of 660 elements and ranges between –1 and 1. Subsequently, the feature vectors were individually fed to a support vector machine classifier with a linear kernel to investigate the different extraction methods (Oskoei et al., 2009; Putze et al., 2014; Li et al., 2018). Here, k-fold cross-validation (*k* = 10, 80% training data, 20% testing data) was applied to subject-independent input data stemming from a random selection across the entire dataset. To calculate subject-specific results, an individual SVM classifier for each subject was trained on the combined VAR-PSD-DWT data. Here, similar trial selection and k-fold cross-validation approaches were used as mentioned earlier.

Finally, a statistical analysis on the difference between visual-only vs. audio-visual k-folds classification results was performed using a Welch’s *t*-test with a 5% significance level. The evaluation is based on the accuracy performance for all folds. The *t*-test assumes that both ensembles are sampled from a normally distributed dataset with unequal variance.

## Results

### Combined Visual Stimuli

**FIGURE 5:**
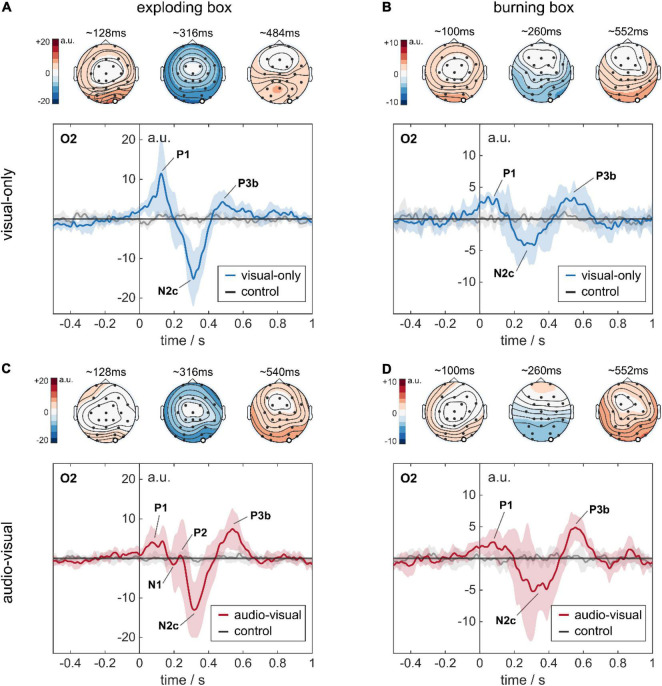
Stimulus-response to complex audio-visual stimuli. All four temporal plots represent the EEG response at the occipital channel O2. The temporal signals are shown as the global average across eight subjects. The standard deviation indicates the variability between the subject-specific average responses. The topoplots represent the global average amplitude distribution across the scalp at three different time points (120, 320, and 540 ms), indicating P1, N2c, and P3b. **(A)** Response to an explosion in a visual-only experiment. **(B)** Response to a burning box in a visual-only experiment. **(C)** Response to an explosion in an audio-visual experiment. **(D)** Response to a burning box in an audio-visual experiment. Note the different y-scale for the exploding and the burning box in the temporal plots.

The explosion and the ignition event are implemented as a combination of visual effects, see videos in Supplementary Material. Thus, we first want to study the neuronal response to such a combinational visual input. For instance, the explosion was mimicked by an upwards flying box and a bright white spherical wave starting at the box and rapidly propagating through space until the entire field of view is filled. Then, the white flash faded out, the box fell downwards until it disappeared at the floor, and the scene stayed blurry until all smoke had vanished. In total, the entire explosion event lasted ∼2 s. Hence, we expect the explosion event to be a spatio-temporal mix of different effects leading to an early visually evoked potential (VEP) induced by the flash at the onset and an event-related potential (ERP) in response to the change of the visual scenery. The global responses to the visual-only exploding and burning box are depicted in Figures 5A,B, respectively.

As visualized in Figure 5A for channel O2, we found deviations at different time instances in the global average response for an explosion compared to the control condition. First, there was a positive rise in amplitude (P1) at O2 in Figure 5A, which started at stimulus onset and peaked with 11.5 ± 9.9 a.u. at ∼125 ms. Then, a negative dip followed, beginning at ∼200 ms and peaking at ∼310 ms to –15 ± 6.9 a.u. Subsequently, a smaller positive rise was observed until a plateau of 4.3 ± 2.8 a.u was reached at ∼430 ms, which decayed slowly afterward. This finding was robust across trials, as the trial colormaps for a single subject show in Supplementary Figure 2. The high standard deviations in the global response, especially for the first peak P1, were caused by the subjects’ large variability in terms of latency and amplitude, as depicted in Supplementary Figure 3. The first rise in amplitude for O2 was also present at the entire parietal-occipital lobe, but with higher amplitude over the primary visual cortex, see topoplots in Figure 5A and the average response for all channels of a single subject in Supplementary Figure 4.

In contrast to the explosion, the burning box (see videos in Supplementary Material) is designed as a progressive rather than a sudden event. Furthermore, it is modeled as less severe since the flames gradually evolve originating at the traveling box. The burning box stimulus was terminated when the box disappeared in the waste container after discarding. The global response to a burning box is visualized in Figure 5B. Here, we find a pattern similar to the explosion – a small P1 between 50 and 100 ms, then a N2c at ∼280 ms, followed by a P3b at ∼520 ms.

### Additional Acoustic Stimuli

As real-world events naturally lead to a combination of visual and auditory cues, we further investigated the influence of additional sounds that match the visual experience in the experiment. To this end, background noise (42 dBA SPL) related to the running conveyor belt was implemented. Furthermore, the explosion and the burning box events were synchronized with suitable audio signals (sounds see Supplementary Material). Here, we complied with the hierarchical approach and implemented different loudness levels for the explosion and the burning box event. The explosion audio signal had a peak level of ∼65 dBA and faded slowly toward the conveyor belt noise floor, correlating with the visual impression. The burning box audio stimulus consisted of a transient signal (lighting a match) that reached a steady state of 50 dBA (fire) until the subject discarded the box. Apart from the additional sound, the experiment was the same as previously described. The global responses to the audio-visual exploding and burning box are depicted in Figures 5C,D, respectively.

In case of an explosion, five characteristic fluctuations at O2 are visible: a positive peak with ∼4 a.u. between 70 and 140 ms (P1), two small-amplitude peaks around 220 ms, followed by a prominent negative peak with −13.0 ± 7.1 a.u. at 320 ms (N2c), and a subsequent positive peak with 7.4 ± 5.2 a.u. at ∼530 ms (P3b). The global response to a burning box with additional auditory cues is shown in Figure 5D. Here, three peaks, P1 with 2.6 ± 2.1 a.u. at ∼80 ms, N2c with –4.4 ± 3.9 a.u. at ∼330 ms and P3b with 4.9 ± 2.0 a.u. at 550 ms are visible, similar to the fluctuations in Figure 5B.

### Offline Classification

Since experiments based on virtual reality nowadays offer a great tool to study the applicability of BCIs, we lastly investigate the detectability of events based on visual-only and audio-visual input. This is particularly interesting, as real-world training data is not always easily accessible – especially if the event is rare and/or severe. Moreover, the implementation of multiple modalities in VR settings can be challenging as well. Thus, we aim to evaluate if the classifier that uses bimodal training data is outperforming the classifier based on unimodal input only. To this end, we tested different feature extraction methods – variance-based (VAR), power-spectral density-based (PSD), and discrete-wavelet-transform based – and performed an offline classification using support vector machines on a subject-independent dataset. Here, all subjects’ data was merged to randomly select training and cross-validation trials afterward. The VAR method calculates the variance of four different windows containing the response in the P1-, the N2c- and the P3b-part, and the entire epoch as shown in Figure 5. The PSD method analyzes the power within the frequency band of 1–30 Hz. In the DWT method, we used a Daubechies mother wavelet to decompose the signal. Additionally, all three methods were combined by concatenation into a single feature vector (DVP) and assessed. The performance of the methods was evaluated with three indicators: (i) the average accuracy across folds indicating the overall model performance, (ii) the average specificity indicating the model performance toward detecting the control condition, and (iii) the average sensitivity that represents the model performance toward detecting the stimulus. The offline detection results are shown in Table 1.

**TABLE 1:** Classification results for the subject-independent dataset.

		Exploding box		Burning box	
		Visual-only	Audio-visual	Visual-only	Audio-visual
Variance method (VAR)	accuracy/%	86.18	87.09	74.01	76.89
	specificity/%	86.5	89.08	79.85	79.27
	sensitivity/%	86.09	85.40	68.39	76.3
Power-spectral density method (PSD)	accuracy/%	82.16	85.58	67.56	76.42
	specificity/%	83.90	87.58	72.6	80.47
	sensitivity/%	80.83	83.02	61.91	72.6
Discrete wavelet transform method (DWT)	accuracy/%	91.16	90.82	78.45	78.73
	specificity/%	90.07	92.36	78.65	79.55
	sensitivity/%	91.70	89.7	79.46	78.66
Feature Fusion Method (DVP)	accuracy/%	**94.56**	**92.92**	**80.78**	**86.36**
	specificity/%	96.10	94.25	84.22	89.47
	sensitivity/%	92.84	91.71	78.32	84.25

*A support vector machine with a linear kernel was used to detect either the exploding or the burning box with respect to the control condition. The results are provided as mean across 10 folds. The Bold values represent the highest achieved accuracy.*

## Discussion

In the following, we will first discuss the neuronal activity in response to the combinational visual stimuli of an explosion and burning box (see Section “Combined Visual Stimuli”). Afterward, the changes in neuronal activity for experiments with additional auditory cues are presented in Section “Additional Acoustic Stimuli”. Focusing on an industrial BCI application, we lastly compare in Section “Offline Classification” the detectability of an explosion or ignition event based on different feature extraction methods using a support vector machine classifier.

### Combined Visual Stimuli

For the explosion box stimulus, we assign this first response P1 to a VEP stemming from a sudden change in light intensity (Connolly and Gruzelier, 1982; Lines et al., 1984; Creel, 1995; Kazai and Yagi, 2003; Sharma et al., 2015; Guo et al., 2019). Further, we associate the negative peak at ∼310 ms with the N2c component of an ERP-response, as it is distributed across the occipital/posterior region (see Supplementary Figure 4A). The N2c component is generally related to visual attention and the processing of stimulus characteristics, which aligns with our expectations of an early primary reaction (P1) and a later activity that reflects the interpretation of the visual scene (N2c and further peaks) (Ritter et al., 1979, 1982; Folstein and Petten, 2008). Lastly, we identify the positive response at ∼430 ms to be a late P300 signal being evoked by the oddball paradigm. Here, the processing in the visual cortex leads to a delayed response, called P3b, which is usually observed after an N2c component (Comerchero and Polich, 1999; Stige et al., 2007). Consistent with other published work, (Katayama and Polich, 1998; Comerchero and Polich, 1999; Stige et al., 2007; Folstein and Petten, 2008) we observed the P3b component to be higher in the posterior region than in the anterior region of the brain, see topoplots in Figure 5A and Supplementary Figure 4A as well.

In the burning box stimulus, the absolute amplitudes are notably reduced to a range of approx. ± 5 a.u., reflecting the lower degree of severity and/or lower attention accumulation compared to the explosion. Interestingly, the P1 amplitude for the burning box was in the same range as its N2c-P3b complex, which is in stark contrast to the explosion stimulus, where the P1 was significantly higher than the P3b. This difference might be firstly explained by the gradual increase of fire, secondly by its bounded extent, and thirdly by the red-orange color scheme of the fire animation compared to a full-screen white flash for the explosion.

The high standard deviations for both global responses can be explained by significant differences in amplitude and – even more critical – latencies across individual subjects (see Supplementary Figures 3A,B). Here, the response variation might also be affected by adaptation and/or the subjects’ engagement and focus throughout the experiment. In summary, we observed a similar neuronal activity – a combination of an early visually evoked potential (P1) and a delayed event-related potential (N2c-P3b complex) – in response to our virtual explosion and burning stimuli. Here, the degree of severity is reflected in the signal amplitudes, leading to a generally reduced response for the burning box compared to the explosion. Both events, however, showed clearly differentiable global average responses compared to the control condition where the box simply travels along the pathway.

### Additional Acoustic Stimuli

In case of an explosion, we find the characteristics of N2c and P3b to be stable, yet their latencies and amplitudes differ (see Supplementary Figure 5A). Surprisingly, the VEP P1 is reduced by a factor of ∼3, whereas the N2c component is similar in amplitude. The P3b component is delayed by ∼70 ms and increased by a factor of ∼2. Consequently, the additional sound had two effects, the primary visual cue is drastically suppressed, and the ERP components are robust (N2c) or enhanced and delayed (P3b) compared to the visual-only findings. Whereas the suppression of the VEP response P1 are suprising, the ERP enhancement seems plausible, as the additional sound provided congruent supplementary information to the subjects’ visual impression. Furthermore, the enhanced N2c signals could also be attributed to increased attention during the experiment since participants (that took both experiments in an initial pilot study) reported the audio-visual experiment to be more engaging in the burning box stimuli. Lastly, two new fluctuations around 220 ms appeared in the global averages, see Figure 5C. Therefore, in line with our hypothesis, the additional small-amplitude peaks could be interpreted as the N1 and P2 components of a strongly enhanced ERP and were not caused by the additional auditory cues. Generally, the N1 and P2 fluctuations of an ERP can be assigned to sensation-seeking behavior, thus reflecting a stronger focus of the participants (Sur and Sinha, 2009). A closer look at the individual responses (Supplementary Figures 5A, 6A) reveals the presence of N1 and P2 in 6 out of 8 subjects that participated in an audio-visual experiment. Surprisingly, the additional P2 is in most cases in the same amplitude range as the visually evoked P1 (see Supplementary Figure 6A), which is not visible in the global averages due to latency differences across subjects. However, we found N1-P2 components also in the visual-only experiment for some subjects (see Supplementary Figure 3A), yet with smaller amplitude compared to an audio-visual experiment. Thus, we conclude that the additional peaks most probably stem from the ERP, which might be altered in amplitude by attention, focus, severity, and congruent input.

The global response to a burning box with additional auditory cues compared to the visual-only experiment, we find the P1 component also to be suppressed by a factor of ∼1.3. However, the N2c and the P3b components are again enhanced by a factor of ∼1.25 and 1.5, respectively. Additionally, we also observed a delayed ERP response. This result is in line with the previous findings for the explosion. Moreover, large standard deviations around 300 ms indicate the presence of additional small-amplitude peaks as well, which is supported by inspecting the individual responses in Supplementary Figures 5B, 6B.

Statistical analysis based on Welch’s *t*-tests revealed a significant amplitude difference in the mean responses for the P1 (*p* = 0.0393) in the case of the explosion stimulus, and further for the N2c (*p* = 0.0412) in case of a burning box at channel O2. However, the P3b component for both conditions did not yield statistical significance as we calculated *p* = 0.0764, *p* = 0.0704 for the exploding box and burning box, respectively. Moreover, all other differences in amplitude and latencies did not provide statistical significance, which can be also explained by the small dataset and large deviations across subjects (see Supplementary Table 1).

In summary, we noticed two different effects on the neuronal responses if additional matching auditory cues were present (see Supplementary Figure 7). Firstly, different levels of severity – explosion versus burning box – were again visible as differences in amplitude. Consistently for both stimuli we found the VEP or primary reaction in the visual cortex to be diminished, whereas the ERP components N2c and P3b were enhanced by the sound. Moreover, two other fluctuations, N1-P2, occurred around 220 ms, which we assign to ERP components prior to the large-amplitude peaks N2c and P3b.

Based on our observations, we conclude that additional auditory cues lead to a suppression of the VEP by inhibitory pathways. This was surprising, as we did not expect the sound to induce changes in the early processing stages of primary visual information. However, recent studies shed light on the complex interplay of the neuronal processing of multisensory input (Driver and Spence, 2000; Calvert, 2004; Marchant and Driver, 2013). Indeed, it has been demonstrated that there is “crosstalk” between modality-specific pathways in the associative cortex (Calvert, 2001; Hidaka and Ide, 2015) as well as the primary sensory cortices (Talsma et al., 2007; Senkowski et al., 2011) leading to an early audio-visual integration (Driver and Noesselt, 2008; Wang Y. et al., 2008; Iurilli et al., 2012; Ide and Hidaka, 2013; Hidaka and Ide, 2015). In line with our data, other groups demonstrated e.g., a decreased fNIRS response in the visual cortex (Wiggins and Hartley, 2015) as well as a suppressed visual perception (Hidaka and Ide, 2015) when sound is presented in a spatially and temporally consistent manner. However, we did not only observe the suppression of the primary reaction in the visual cortex (VEP) but also an enhancement and a delay of the following ERP response for additional sound. This could be caused due to differences in the population for both experimental conditions. However, we experienced the phenomenon on single subjects in pilot studies to be robust. In fact, various effects – both, facilitatory and inhibitory – have been reported for multimodal audio-visual input (Stein et al., 1996; Shams et al., 2005; Hidaka et al., 2009; Meredith et al., 2009; Romei et al., 2009; Gleiss and Kayser, 2013). For instance, it was shown that a multimodal (e.g., visual, acoustic, and tactile) compared to unimodal (visual) stimulation can lead to a drastic enhancement of the P300 signal. (Wang W. Y. et al., 2012; Marucci et al., 2021). Interestingly, an additional delay of the ERP, as visible in our data, was not explored. One could attribute the ERP delay to originate from weak inhibition effects that eventually lead to longer responses (Wang W. Y. et al., 2012). Yet, we found the ERP responses to be more prominent and robust in the audio-visual experiment. Thus, we conclude that a multimodal stimulus leads to an increased certainty about visual perception. Especially in the case of the burning box, where the unimodal visual perception is less clear, the additional (informative) sound supports the understanding and discrimination of the scene (Stein et al., 1996; Talsma et al., 2007; Senkowski et al., 2011; Gleiss and Kayser, 2013).

### Offline Classification

Last, we investigated the effect of multimodal stimuli on their classifiability by using offline classification. In this way, we are able to test different extraction methods in a time effifient manner and apply our findings to online classification schemes.

As expected, detecting an explosion is less challenging than detecting a burning box; see absolute values of all criteria in Table 1, both in a visual-only and in an audio-visual experiment. Here, we observe significant amplitude differences between the explosion and the burning box responses. The best single-method detection performance for both a visual-only and audio-visual experiment was achieved with the DWT approach (e.g., 91.16 % for an explosion and 78.45 % for the burning box in a visual-only experiment). In contrast, PSD and VAR-based detection performances were substantially lower. This can also be partially explained by correlations between the mother wavelet of the DWT and the neuronal response (Samar et al., 1999). Furthermore, the concatenation of all three feature vectors (DVP) led to an improvement in both conditions (visual-only and audio-visual) for both stimuli compared to DWT. Again, this was partially expected since a larger feature vector can provide more information to the classifier. In the subject specific classification, we achieved an average detection accuracy of 96.06 and 79.96 % for the explosion and burning box, respectively.

**FIGURE 6:**
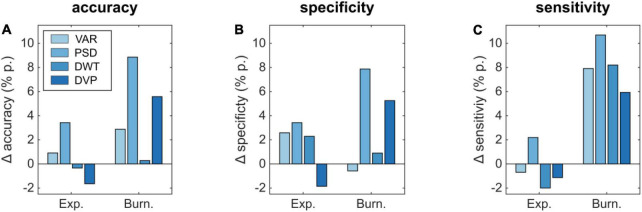
Differences in classification performance parameters **(A)** accuracy, **(B)** specificity, and **(C)** sensitivity for an audio-visual compared to a visual-only experiment.

The effect of additional auditory cues on the detectability based on different features is shown in Figure 6. Here, the accuracy for the explosion (Figure 6A) improves by 0.91 and 3.42% p. for VAR and PSD, whereas the DWT and DVP-based performance decreased by –0.34 and –1.64% p., respectively. In case of the burning box, additional auditory cues lead in all cases to an improvement, most prominent for the PSD (8.86% p.) and the combined DVP (5.58% p.). Similarly, the specificity and sensitivity for the burning box are also increased in all but one method, if additional auditory cues are present. In case of the explosion, there is not always an improvement. Mainly if the extraction method relies on the strong P1-contribution in the visual-only experiment (VAR, DWT, and DVP), the performance is slightly decreased in case of additional sound. Similar to ensemble values, we observed a slightly decreased subject-specific classification accuracy (based on DVP) of 94.53% for the audio-visual explosion compared to the visual-only. Again, the burning box led to opposite results. Here, the accuracy increased to 85.18%.

Statistical analysis based on Welch’s *t*-tests revealed a significant classification accuracy difference in case of the burning box stimulus for the VAR, PSD, and DVP feature extraction methods (*p* = 0.0044, *p* < 0.001, *p* = 0.0050). Moreover, the exploding box stimulus classification accuracy did not yield statistical significance, which can be explained by the small increase or decrease in performance and the overlapping standard deviation between folds.

The results for the burning box highlight that multimodal input can lead to more robust and enhanced ERP patterns that guarantee an enhanced classification performance. In fact, future real-world detection tasks will resemble most likely the burning box-type of situation, where isolated sensory inputs are less severe, hence, attention-grabbing. Here, the consistent multisensory experience leads to a stronger attentional shift and an increased certainty about the (complex) situation. Consequently, we expect that BCIs trained on multimodal input will show an enhanced classification performance in real-world settings compared to BCIs that consider only unimodal input.

## Conclusion

Within this work, we studied neuronal responses to two complex stimuli – an exploding box and a burning box – with different perceived severities. The response consisted of a strong early VEP component and a smaller delayed ERP complex in the explosion. The burning box evoked a similar pattern consisting of a minor VEP component and the following ERP complex, but significantly smaller amplitudes than the explosion. Thus, the effect of different severity levels was reflected in the signal amplitudes. Surprisingly, the effect of additional auditory input was not consistent for all response components. Most prominently, for the exploding box, the initial VEP was significantly suppressed in the audio-visual experiment. Moreover, we observed additional small-amplitude peaks around 220 ms after stimulus onset, which we attribute to the early small-scale ERP fluctuations N1 and P2. Hence, we conclude that congruent multimodal sensory input leads to greater attention and/or a more confident evaluation of the input data, resulting in a robust ERP signal.

In summary, experiments in a virtual environment offer great potential to test the potential of BCIs in different applications. However, stimuli that mimic real-world situations elicit complex neuronal patterns that highly depend on the exact stimulus and environment. As shown in this work, step-by-step VR-EEG studies provide means to bridge the gap from experiments under “clean” lab conditions toward specifically tailored BCI systems. Here, we demonstrated that inhibition and facilitation effects alter the signal for a combined audio-visual input. Based on a SVM classifier, we showed an improvement in the detectability of a bimodal audio-visual stimulus compared to a unimodal visual input. As real-world experiences are multimodal by nature, the early integration of multisensory input has a significant impact on the design of future VR BCI studies.

## Data Availability Statement

The raw data supporting the conclusions of this article will be made available by the authors, without undue reservation.

## Ethics Statement

The studies involving human participants were reviewed and approved by Ethics Commission of the Technical University of Munich. The patients/participants provided their written informed consent to participate in this study.

## Author Contributions

GA, LW, PR, and BW designed the study. GA, LW, and SM carried out the experiments. WH and LH helped with the experimental setup. LW and GA wrote the manuscript with support from PR, WH, and BW. All authors provided critical feedback.

## Conflict of Interest

The authors declare that the research was conducted in the absence of any commercial or financial relationships that could be construed as a potential conflict of interest.

## Publisher’s Note

All claims expressed in this article are solely those of the authors and do not necessarily represent those of their affiliated organizations, or those of the publisher, the editors and the reviewers. Any product that may be evaluated in this article, or claim that may be made by its manufacturer, is not guaranteed or endorsed by the publisher.
