# Supplementary material for: Influence of Auditory Cues on the Neuronal Response to Naturalistic Visual Stimuli in a Virtual Reality Setting
Source: Front Hum Neurosci. 2022 Jun 2;16:809293. doi: 10.3389/fnhum.2022.809293 (PMC9201822; doi:10.3389/fnhum.2022.809293)
Supplement: Supplementary file 3 [file Data_Sheet_1.docx]

Supporting Information To

‘Influence of auditory cues on the neuronal response to naturalistic visual stimuli in a virtual reality setting’

**George Al Boustani^1,†^ , Lennart Jakob Konstantin Weiß^1, †^, Hongwei Li^2^, Svea Marie Meyer^1^, Lukas Hiendlmeier^1^, Philipp Rinklin^1^, Bjoern Menze^2^, Werner Hemmert^3^, Bernhard Wolfrum^1,*^**

^1^ Neuroelectronics – Munich Institute of Biomedical Engineering, Department of Electrical and Computer Engineering, Technical University of Munich, Boltzmannstraße 11, 85748 Garching, Germany

^2^ Department of Quantitative Biomedicine, University of Zurich, Switzerland; and Department of Informatics, Technical University of Munich, Germany

^3^ Bio-Inspired Information Processing – Munich Institute of Biomedical Engineering, Department of Electrical and Computer Engineering, Technical University of Munich, Boltzmannstraße 11, 85748 Garching, Germany

* Corresponding Author: [bernhard.wolfrum@tum.de](mailto:bernhard.wolfrum@tum.de)

^†^ These authors contributed equally to this work.

# Effect of Common Average Re-Referencing (CAR)


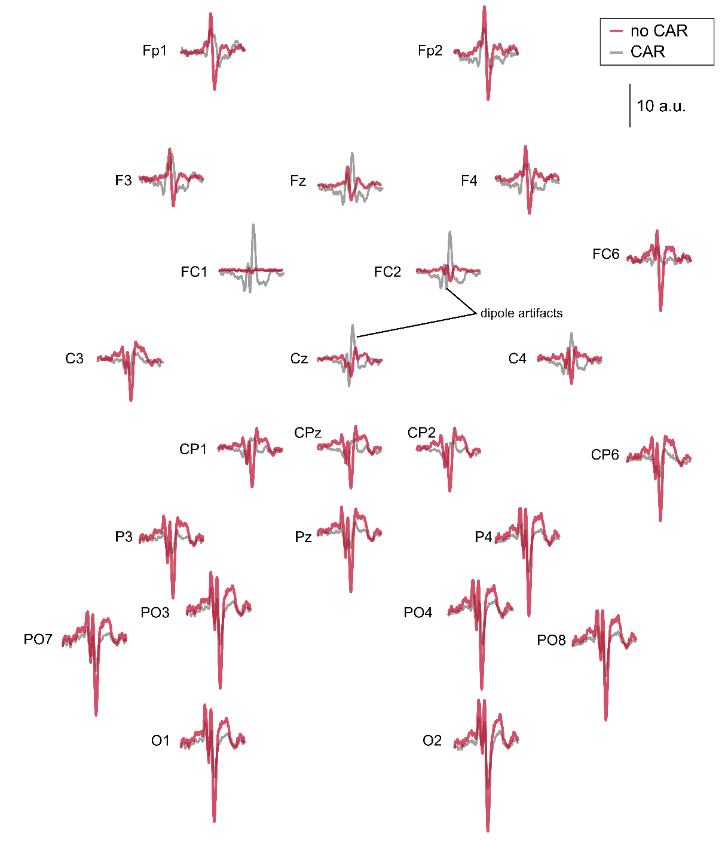


**Figure S1**: Average Response of a single Subject with respect to an exploding box in an audio-visual experiment with and without common-average re-referencing (CAR). In CAR, dipole artifacts in the central-frontal region arise due to the synchronous activity affecting the mean signal in a high number of channels in the occipital lobe.

# Responses of Individual Subjects

#
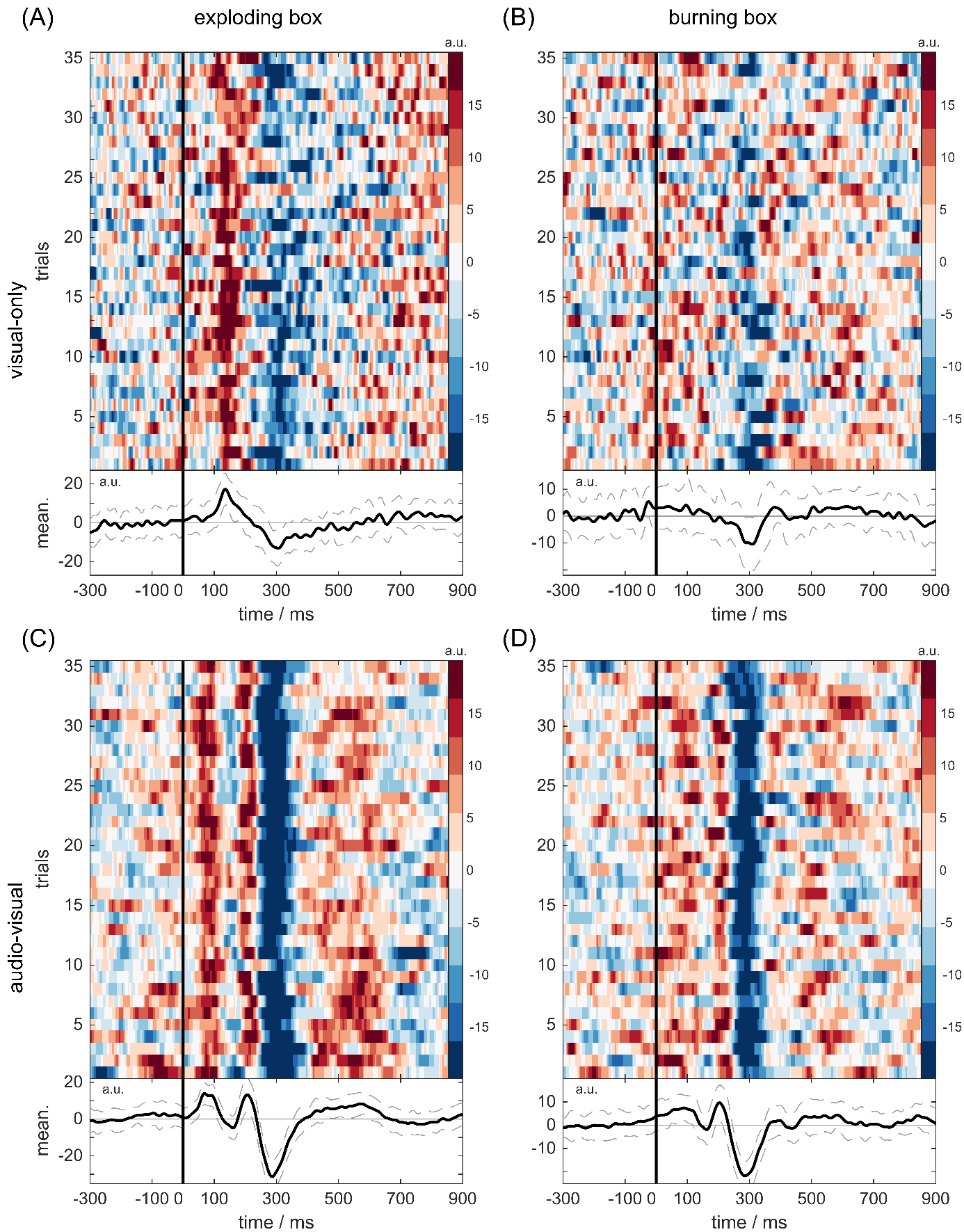


**Figure S2: Individual Stimulus Responses to Complex Audio-visual Stimuli.** All four temporal plots represent the EEG responses at the occipital channel O2. The recordings for visual-only and audio-visual experiments stem from different subjects. **(A)** Response to an explosion in a visual-only experiment. **(B)** Response to a burning box in a visual-only experiment. **(C)** Response to an explosion in an audio-visual experiment. **(D)** Response to a burning box in an audio-visual experiment. Note, the different y-scale for the exploding and the burning box in the temporal plots. The trial colormaps feature the same color range to highlight the severity difference between both stimuli.

# Individual Responses in an Visual-Only Experiment


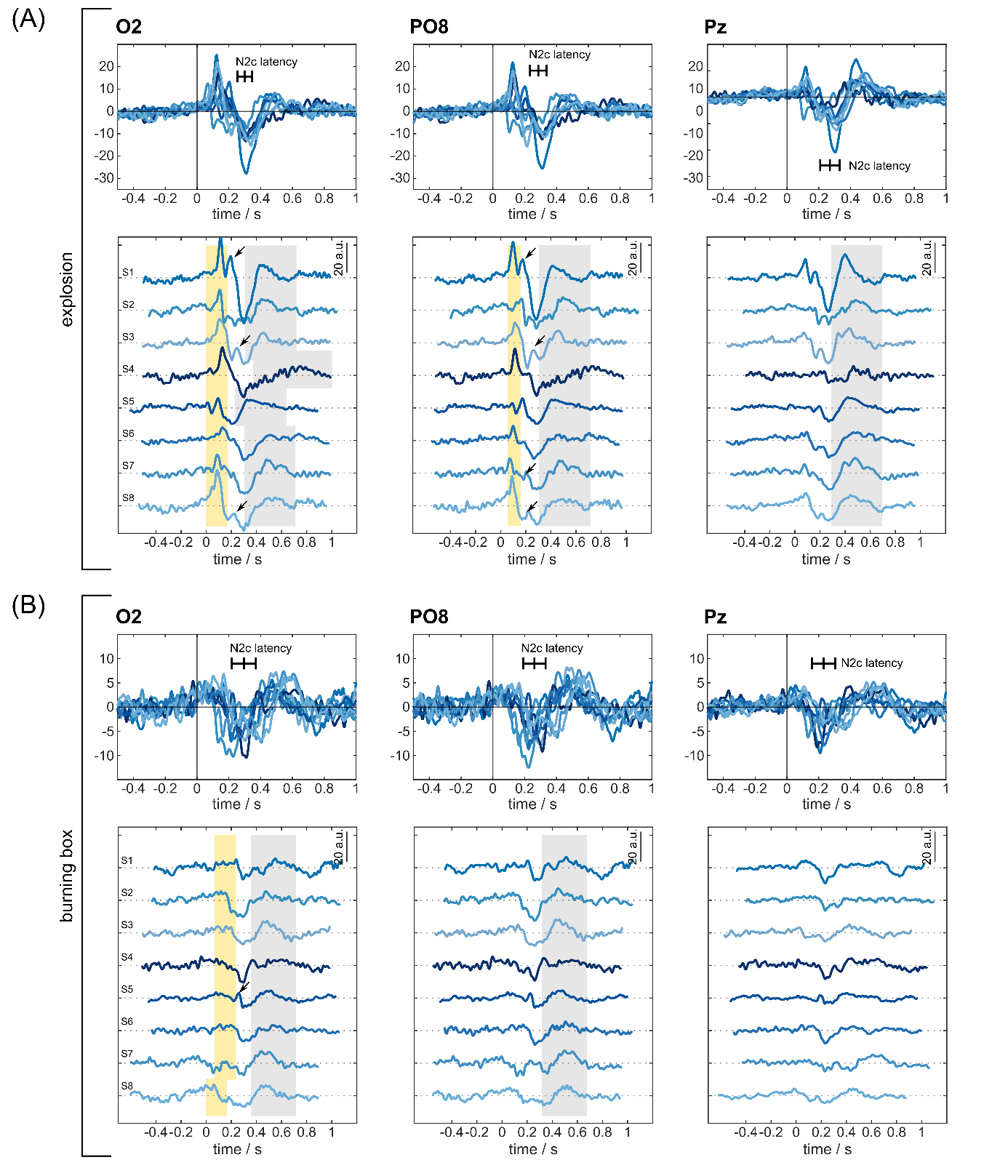


**Figure S3**: Average activity at channels O2, PO8, and Pz (n ≥ 38 trials) of all subjects in the study in response to **(A)** an explosion event and **(B)** a burning box event. The top row shows the overlays of all subject mean, and the bottom row shows the averages being time-adjusted with respect to the N2c minima. Yellow and grey shadings indicate the P1 (VEP) and P3b (ERP) components. Arrows highlight additional positive fluctuations P2 before the large-amplitude N2c-P3b complex.

# Temporal Averages of All Channels for a Single Subject in a Visual-Only Experiment


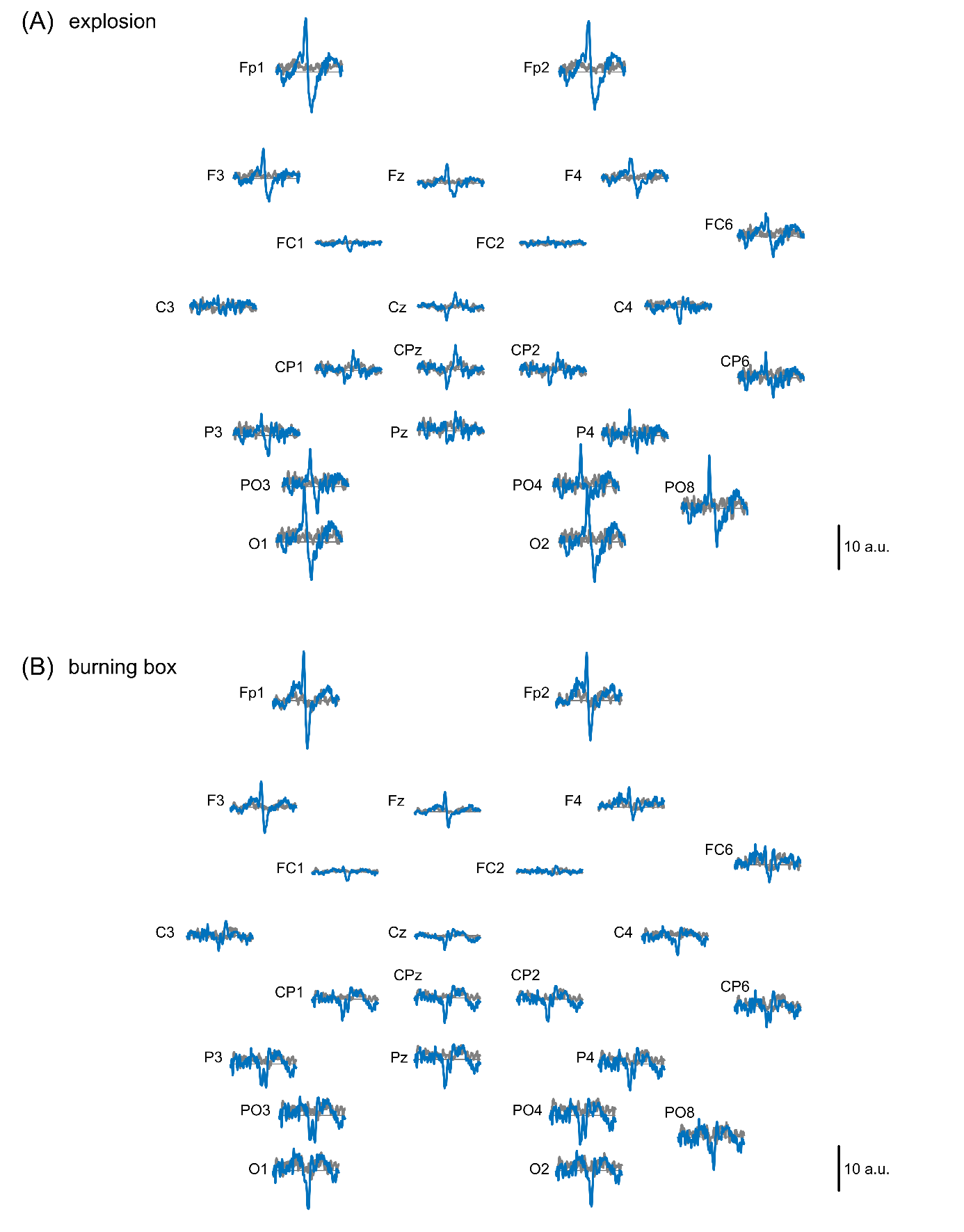


**Figure S4:** Average response for all channels of a single subject in case of a complex visual-only stimulus. **(A)** Response to an explosion. **(B)** Response to a burning box. They gray lines show the average activity for the control condition.

# Individual Responses in an Audio-Visual Experiment


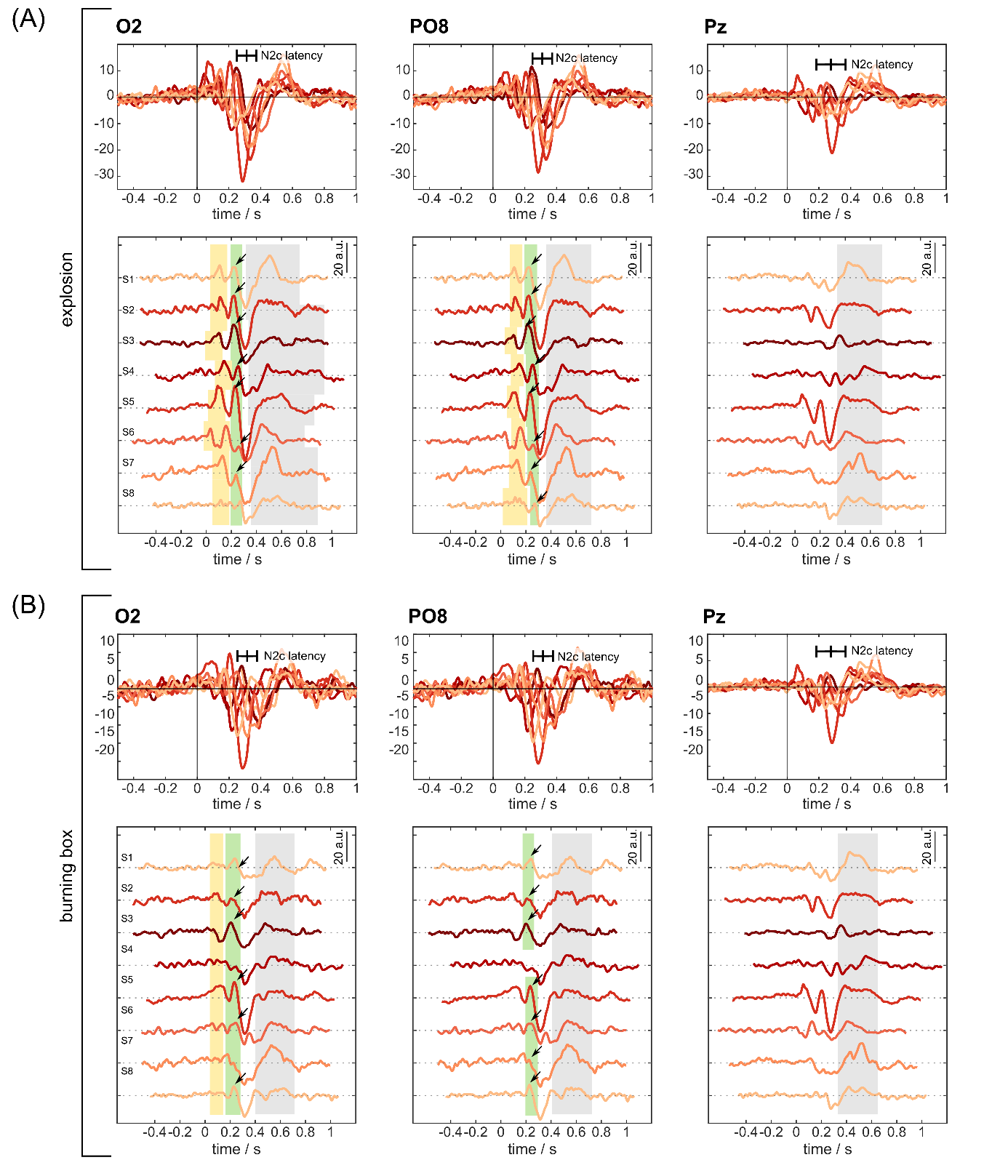


**Figure S5**: Average activity at channels O2, PO8, and Pz (n ≥ 38 trials) of all subjects in the study in response to **(A)** an explosion event and **(B)** a burning box event. The top row shows the overlays of all subject mean, and the bottom row shows the averages being time-adjusted with respect to the N2c minima. Yellow and grey shadings indicate the P1 (VEP) and P3b (ERP) components. Arrows and the green shading highlight additional positive fluctuations P2 before the large-amplitude N2c-P3b complex.

# Temporal Averages of All Channels for a Single Subject in an Audio-Visual Experiment


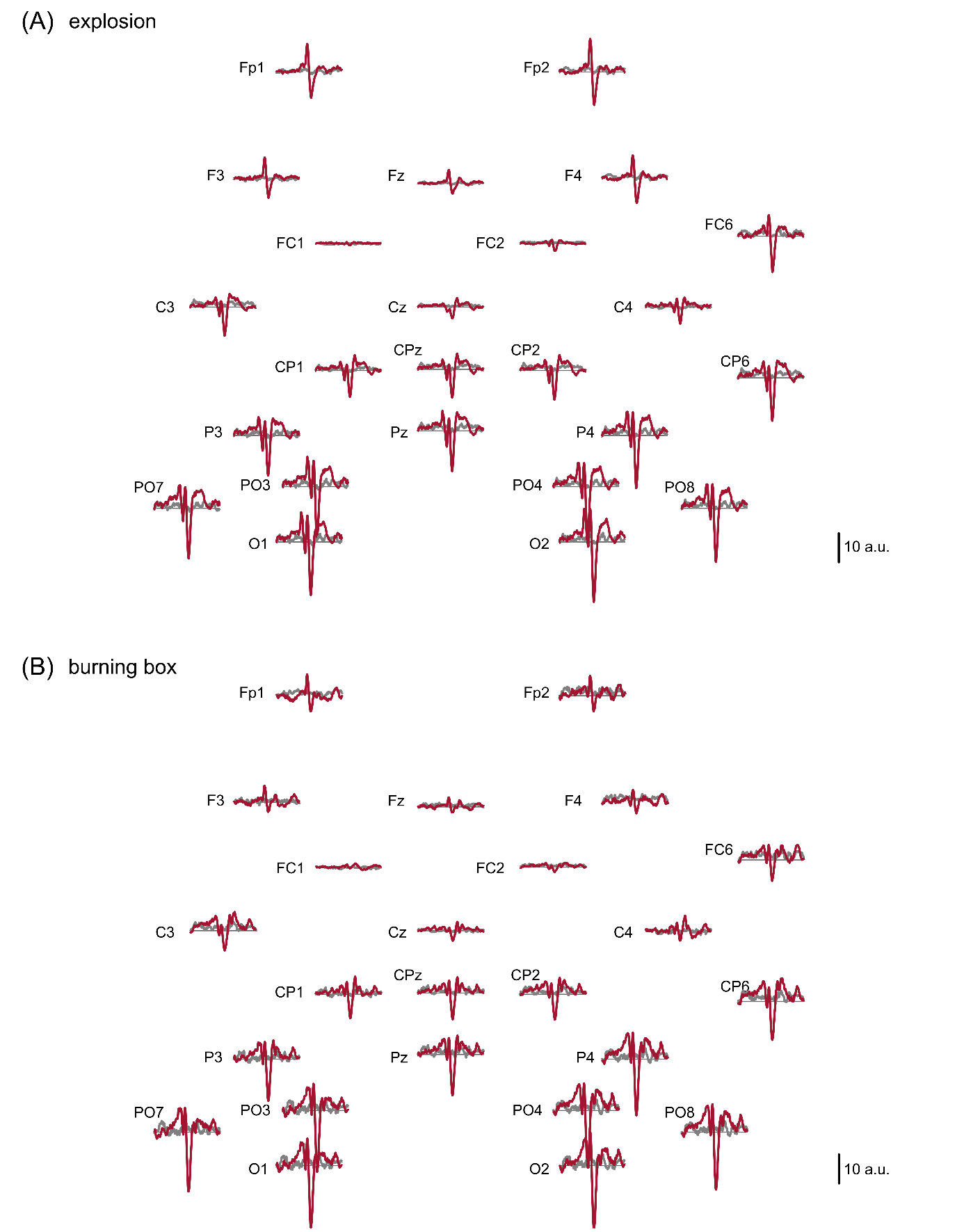


**Figure S6:** Average response for all channels of a single subject in case of a complex audio-visual stimulus. **(A)** Response to an explosion. **(B)** Response to a burning box. They gray lines show the average activity for the control condition.

# Effect of Additional Acoustic Cues


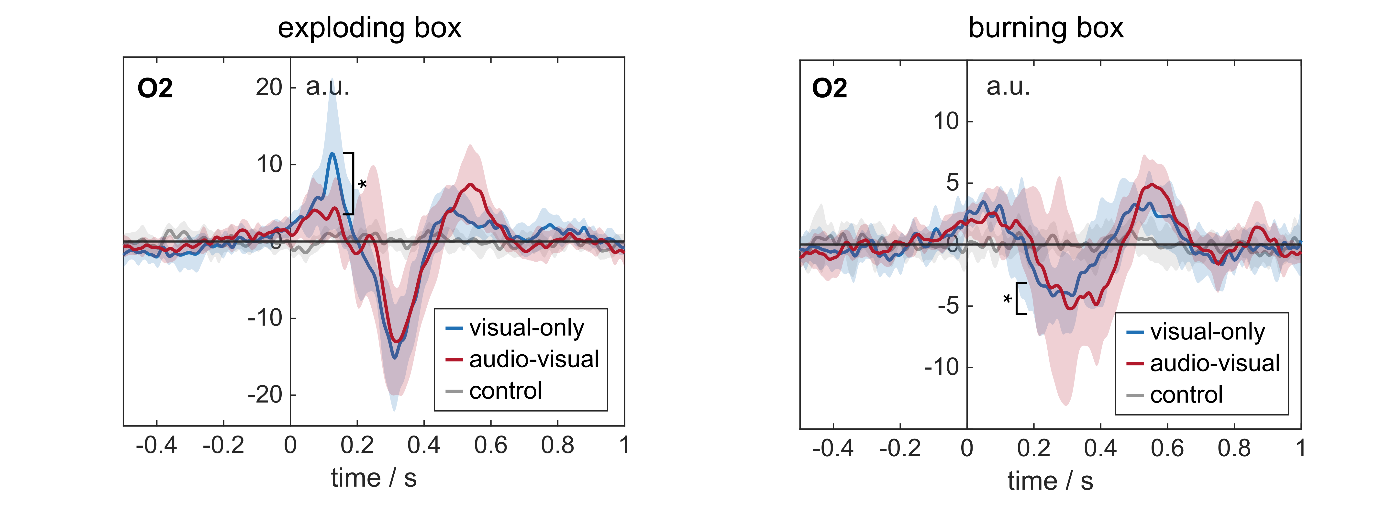


**Figure S7**: Individual Stimulus Responses to Complex Audio-visual Stimuli. All four temporal plots represent the EEG responses at the occipital channel O2. Significant differences in amplitude are indicated with an asterix.

# Sound Sources

The ignition sound (ArrowIgnite01_SteamVRAsset_sounds) was taken from the steam VR asset collection. The explosion sound (https://freesound.org/people/deleted_user_5405837/sounds/399303/) and the conveyor belt noise (https://freesound.org/people/freemaster2/sounds/172350/) were collected from the open source sound library freesound.org.

# Statistical Analysis

**Table S1:** **t-test statistical analysis results in p-values.** The statistical analysis was performed using a t-test with a 5% significance level, to reinforce enhancement and suppression effects caused by the multimodal integration. The evaluation is based on the maximum (VEP, P3b) and minimum (N2c) for each subject’s average at channel O2.

| Exploding Box | | | Burning Box | | |  |
| --- | --- | --- | --- | --- | --- | --- |
| VEP | N2c | P3b | VEP | N2c | P3b |  |
| Visual Only vs Audio-Visual | **0.0393** | 0.2932 | **0.0764** | 0.9007 | **0.0412** | **0.0704** |
